# Facebook addiction and its impact on depression: a cross sectional study

**DOI:** 10.1186/s41043-024-00556-w

**Published:** 2024-06-06

**Authors:** Nahid Salma, Farhaduzzaman Bhuiyan

**Affiliations:** Department of Statistics and Data Science, Jahangirnagar University, Savar, Dhaka, Bangladesh 1342

## Abstract

**Aim:**

Facebook has become a part of daily life for its users and therefore become a very significant factor of mental health. As the number of Facebook users increases exponentially, the problems related to immense use have become more evident and more frequent. Therefore, the study aimed to explore the impact of Facebook addiction on depression among its users.

**Methods:**

An online-based questionnaire was used to collect data from 269 Facebook users of Bangladesh using convenient sampling technique. Bergen Facebook Addiction Scale (BFAS) and Patient Health Questionnaire (PHQ-9) scale was used to assess Facebook addiction and its impact on depression.Ordinal logistic was used to extract the significant variables associated with Facebook addiction and depression.

**Results:**

The result categorized the Facebook users into three categories as : normal (17%), problematic user (52%) and addicted (30.5%). Facebook users were suffering from mild (13.4%), minimal (15.6%), moderate (59.5%), and severe (11.5%) level of depression. Both Facebook addiction and depression were moderately correlated (0.701). Ordinal regression showed, gender $(\beta$ (95% CI) = 0.859 (0.223,1.495)), age ($\beta$ (95% CI) = -2.051(-3.789, -0.313)), residential area ($\beta$ (95% CI) = -0.858(-1.626, -0.09)), occupation ($\beta \left(95\text{\%} \text{C}\text{I}\right)=-0.494(-\text{1.547,0.559})$), time length of Facebook use ($\beta \left(95\text{\%} \text{C}\text{I}\right)=2.288\left(\text{0.324,2.251}\right))$are the significant predictors of Facebook addiction. Wheras, relationship types and occupation are also responsible for depression.

**Conclusion:**

The study showed large portion of facebook users of Bangladesh are suffering from depression. Authors suggest to arrange mental health campaign to promote bounded and legitimate use of facebook and therefore will accelerate the attaining rate of sustainable development goal related to the Bangladesh’s health status.

## Introduction

Since its invention in 2004, Facebook has become a global phenomenon and the trendiest social networking media worldwide (Błachnio et al. 2016). As a media of communication, Facebook is now the largest social networkin site (Zaffar et al. 2015). The purpose behind the Facebook invention was “to make the world more open and connected” by allowing its users to stay in touch (Mihai-Bogdan et al. 2020). Facebook users can keep in touch with family and friends, share personal matters, and make new friends worldwide (Błachnio et al. 2016). With the time it bought many more services and methods these are making this platform more interesting and kepping the user for more amount of time.

The term “Facebook depression” was introduced by the American Academy of Paediatrics (AAP) in 2011 to describe the possible phenomena in which youngsters who spend a lot of time on social networking sites may later display indications of depression (O’Keeffe & Clarke-Pearson 2011). Excessively engaging in human-machine contact is a hallmark of Facebook addiction, a non-chemical addiction (Cerniglia et al. 2017). When someone spends an excessive amount of time on Facebook and begins to exhibit the usual signs of depression, it’s known as Facebook depression (Xie and Karan 2019). As the number of Facebook users increases exponentially, the problems related to immense use have become more evident and more frequent and consequently draw the attention of researchers (Sayeed et al. 2020).

At recent time excess use of Facebook has become a topic of concern regarding its addiction which has a connection to a number of problems that includes anxiety, depression, loneliness and other negative outcomes (Hormes 2016; Marino et al. 2018). Too much time or focus on socal media like Facebook left users with these kind of mental issues (Yoon et al. 2019). However, organisations such as the World Health Organisation and the American Psychiatric Association are still unable to classify it as a recognised mental health illness (Wolniczak et al. 2013; Tang & Koh, 2017). Nonetheless, there is strong evidence that using social media, especially Facebook, can be detrimental to a large number of people and that it resembles behavioural addictions like gaming and gambling in many ways (Alenezi et al., 2023). A total of 2.80 billion individuals using Facebook worldwide. In addition, it -is estimated that over 350 million individuals worldwide meet the medical criteria for addiction (Gaille 2017). Research has indicated that addiction to Facebook stimulates exactly the same brain regions as addiction to drugs (Turel et al. 2014). Moreover, the recent study revealed that the relationship between Facebook use and depressive symptomps also vary due to personality and usage pattern (Rosen et al., 2013).

According to the last update of a popular website named NapoleonCat., till May 2023, about 59 004 100 people in Bangladesh have been using Facebook, which is supposed to be 33.6% of its total population. Additionally, accessing Facebook in the country surfing a smartphone or internet connection is completely free of charge (Al Mamun & Griffiths, 2019). However, Bangladesh has not done much research on Facebook addiction and its association with depression, a newly recognised mental health concern. As far as the current writers are aware, there have only few research on Facebook use in Bangladesh (Al-Jubayer 2013; Jahan & Ahmed 2012; Soron and Tarafder 2015 and Al Mamun & Griffiths 2019). Where none of the first three authors investigated Facebook addiction and depression and the last author investigated from only students. Therefore, this study aimed to explore the effects of Facebook addiction on depression among its users.

## Methods

### Participants

The required sample size was calculated from the following Cochran’s sample size determination formula (Cochran 1977).

$$n=\frac{{z}^{2} p(1-p)}{{d}^{2}}$$

Given that this number yields the largest sample size, the sample proportion of 0.5 was taken (Yasmin et al. 2022). Also, z = 1.96, 6% acceptable margin of error (*d = 0.06*) and confidence interval as 0.05 was considered. Therefore, the required sample size is 267. A convenient Sampling Technique was used and 269 samples was collected and therefore considered for final analysis.

### Study design and procedure

To assess the impact of Facebook addiction on depression among Facebook users of Bangladesh, a cross-sectional study was conducted among Facebook users from April 4, 2023 to May 4, 2023. An online questionnaire (Google Form) was constructed and shared with respondents. Having and using an active Facebook account is the only inclusion criterion for respondents. Bergen Facebook Addiction Scale (BFAS) (Andreassen et al. 2012) was used to measure Facebook addiction and a 9-item Patient Health Questionnaire (PHQ-9,Kroenke et al. 2001) was used to measure depression among Facebook users of Bangladesh. The respondents were well informed about the aim of the study and ensured that their provided information will be kept as a secret. Also, a consent form was provided to them before completing the online questionnaire. Using social media (Facebook, WhatsApp, and Instagram), the online questionnaire was distributed among respondents.

### Measures

#### Bergen facebook addiction scale

To measure the addiction level of Facebook a faculty form University of Bergen developed a scale (Andreassen et al. 2012). This was a collaboration project with the Bergen Clinincs Foundation in Norway. The six items on the BFAS are on a Likert scale with five points (for example, “How often over the past year having you experienced a temptation to use social media further and further?“). and “How frequently did you use social media in the past year to avoid thinking about private issues?” with marks spanning 6 to 30, and varying from very rarely (1) to very often (5). Griffiths (1996, 2005) identified six fundamental components of addiction, which are represented by each item on the list: salience, mood modulation, tolerance, withdrawal, conflict, and relapse. The investigations focused on signs encountered in the previous 12 months. Higher scores correspond to a higher chance of Facebook addiction. Given data on other types of addiction and the fact that the first study lacked cutoff figures, there are two plausible category strategies for troublesome BFAS results. A monothetic scoring system (limit rating: equal to or greater than 3 on all six items) is used by the more traditional strategy, while the more liberal approach uses a polythetic scoring tactics (limit rating: 3 on at least four of the six items) (Andreassen et al. 2012; Andreassen and Pallesen, 2014). Total score was categorized as ≥ 19 = Addicted, 12–18 = Problematic Use, < 12 = Normal User. Prior studies have demonstrated the BFAS’s strong validity and reliability (Atroszko et al. 2018; Brailovskaia et al. 2018). Cronbach’s score for this study was 0.82 which indicates excellent reliability of those scales in this study.

#### Patient health questionnaire

The Patient Health Questionnaire (PHQ-9) comprises nine items that measure depression (Spitzer et al. 1999). Every depression item on the PHQ-9 lists one sign that fits a single DSM-IV diagnosis category for major depressive disorder, such as “To have a lack of enjoyment in doing things,” “Feeling exhausted or having poor enthusiasm,” and “Having difficulties in concentrate.” On a 4-point Likert scale (0 being not at all, 1 denoting several days, 2 representing more than half of the days, and 3 meaning nearly each day), respondents assessed the average incidence of the nine symptoms during the previous two weeks. According to Spitzer et al. (1999), the items use a 4-point Likert rating system. The nine items are combined to provide a screening test (score range 0–27, where 0 reprnbesents no symptoms of depression and 27 represents all symptoms happening almost everyday). where total scores were represented as 0–4 = minimal, 5–9 = Mild, 10–19 = moderate and 20–27 = severe depression (Kroenke et al. 2001). According to earlier research, the degree of internal dependability was deemed suitable and high (Kroenke et al. 2001; Yu et al. 2012). The current study’s Cronbach’s alpha was an excellent of score 0.88.

#### Questionnaire

The questionnaire contains three section. Socio-demographic information of respondents (i.e., gender, age, resident area, family type, relationship status, occupation). Respondent’s information related to Facebook Use (i.e., what kind of internet connection do you use, how long have you been using Facebook, which device do you mostly use for Facebooking, for which purpose do you use Facebook mostly, how frequently do you post on Facebook, what do you post mostly on Facebook, what type of content do you like to see on Facebook, how many hours a day do you use Facebook, approximately how many friends do you have on your social media account, do you post a picture on FB or any other social media, then constantly check for the no. of reactions and comments, do you ever feel your working time has dragged down due to checking your social media feed). The last section contains the questions of BFAS scale and PHQ scale.

### Statistical analysis

Descriptive statistics was conducted to exemplify the basic information of the respondents. Cronbach’s alpha coefficient was used to check the reliability and consistency of study variables. To measure the association between Facebook addiction, depression and other socio-demographic other related variables, chi-square test was used. Finally, Ordinal regression was performed to find out the significant variables associated with Facebook addiction and depression and all the predictor variables. Here, a p-value < 0.05 is used as the significant threshold. Data analysis was conducted using IBM SPSS (Statistical package for social science) for Windows (Version 22.0).

#### Ethical statement and consent to participate

Primary data was used in this study where participants participate completely volunteerly. Prior to survey, all participants were well informed about the objective of this research and were assured about the safety of their provided information. They survey was conducted online, maintaining the rules of Helsinki Declaration about the ethical issue of involving human participants.

## Results

A descriptive analysis was conducted to examine the demographic characteristics and mobile internet usage patterns of the participants, and the size of the sample was 269. The study found that most respondents were female (Table 1), with 150 participants identifying as female and 119 as male. The participants’ ages ranged from 20 to 46, with a mean age of 24.74 years and a standard deviation (SD) of 4.75. Exploring the information, it was observed that most of the respondents (64.7%) belong to the age interval of 20–24, where the mode age was found to be 22 years, holding 52 participants in this specific age. Most of the participants (78.07%) received their first smartphone between the ages of 16 to 20 years. The study also found that the majority of participants were from urban areas (217) as opposed to rural areas (52). The study also examined the family structure, finding that 183 participants belonged to nuclear families while 86 were in joint families. The study discovered that 38 individuals said they solely used mobile internet, 41 said they only used broadband, and 190 said they used both. The vast majority of respondents (190) claimed to use both mobile and broadband, demonstrating that they have access to a variety of internet connectivity options. The study’s findings revealed that more than half of the respondents (60%) were single, whereas many persons (22.3%) were found to be married, and only 47 participants were in a relationship.

Based on the survey results, more than half of the respondents (60.6%) have been using Facebook for more than five years, while around 33% have used it for 2–5 years. A vast majority of the respondents, over 96%, use mobile phones for Facebooking while comparatively only a small portion (3.7%) use desktop or laptop to access it. Regarding the use period, almost half of the respondents (47.2%) use Facebook for 1–3 h per day, and over half (58.7%) use it for entertainment. The majority of respondents (64.3%) have 200–1500 friends on their social media accounts, while 58 (21.5%) of them have more than 1500 friends. Discovering the content that they like to see on Facebook, entertainment-related content (78.8%) is found to be the most popular.

**Fig. 1:**
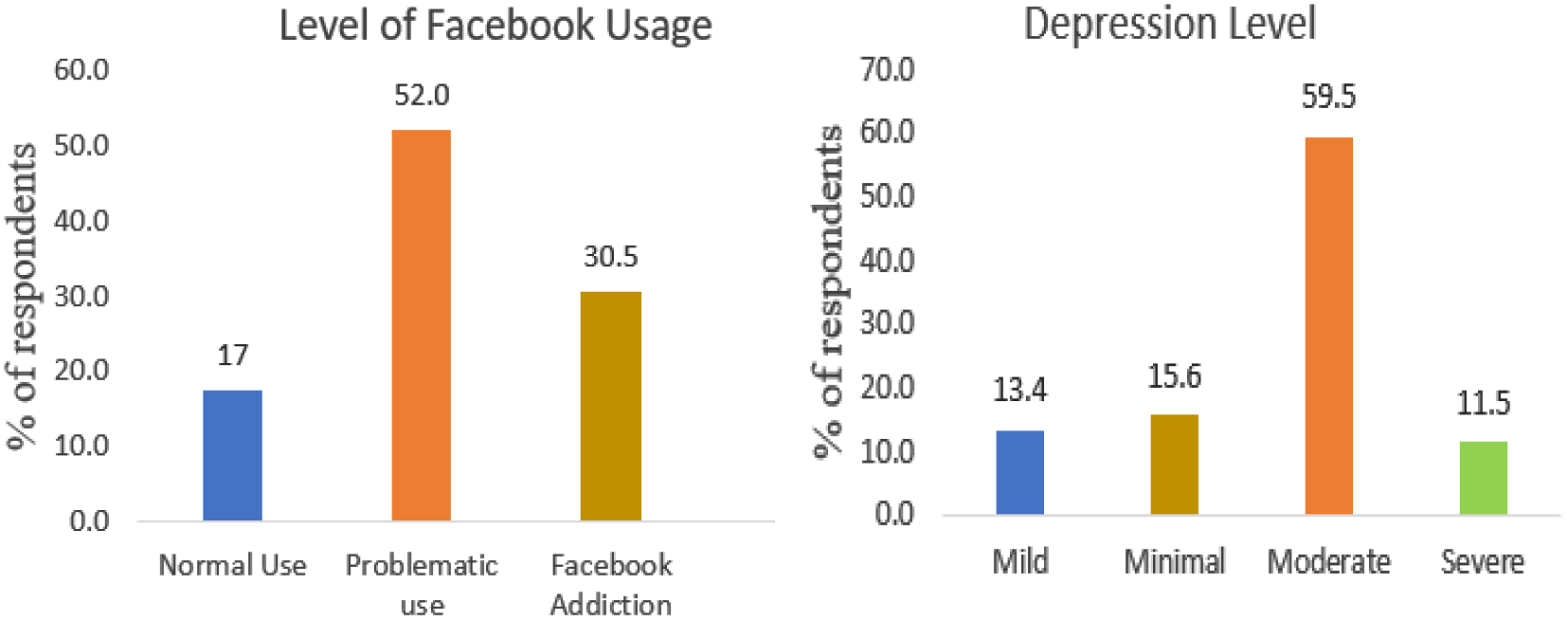
Distribution of facebook users according to facebook usage and depression level

The result in Fig. 1 shows an alarming finding from the responses. Analyzing the scores of BFAS and PHQ scale scores, these come to the front. More than half of the respondents are found that they are in the problematic uses category, and over 30% are Facebook addicted. We also discovered that about 60% of the participants are facing moderate depression, whereas another 11.5% are in severe condition (Fig. 1).

**Table 1:** Demographic characteristics of the respondents

Characteristics	n	%	Characteristics	n	%
Gender			Family Type		
Male	119	44.2	Nuclear	183	68.0
Female	150	55.8	Joint	86	32.0
Age			Occupation		
19–24	174	64.7	Student	199	74.0
25–29	57	21.2	Employed	63	23.4
30+	38	14.1	Other	7	2.6
Relationship Status			Income		
Single	162	60.2	Less than 20000	189	70.3
Married	60	22.3	20000–50000	47	17.4
In a relationship	47	17.5	50000–100000	11	4.1
			More than 100000	22	8.2
Residential Area					
Urban	217	80.7			
Rural	52	19.3			

The results shown in Table 2 depict no association (*p*-value of ${\chi ^2}$= 0.111 > 0.05) between gender and level of Facebook addiction. The study reveals that 37% of males and more than 27% of females are Facebook addicted. The family type also shows a similar scenario of no association (*p*-value of ${\chi ^2}$= 0.210 > 0.05) with the level of Facebook usage. About half of the participants from the urban areas are found in a problematic level of usage of Facebook. More than 90% of the respondents who are in a relationship found problematic use or Facebook addiction. Nearly 35% of students are in Facebook addiction, and only 19% of employed person are in the same condition.

**Table 2:** Association between background characteristics and BFAS scale

Characteristics		Level of Facebook Use			Total	*P*-value
		Normal Use	Problematic use	Facebook Addiction		
Gender	Male	20 (16.8)	55 (46.2)	44 (37.0)	119 (100)	0.111
	Female	10 (8.77)	29 (25.44)	31 (27.19)	114 (100)	
Region	Urban	45 (20.7)	105 (48.4)	67 (30.9)	217 (100)	0.007*
	Rural	2 (3.8)	35 (64.3)	15 (28.8)	52 (100)	
Family Type	Nuclear	54 (16.31)	94 (51.4)	61 (33.3)	183 (100)	0.210
	Joint	28 (15.3)	9 (32.14)	12 (42.86)	86 (100)	
Relationship Status	Single	23 (14.2)	87 (53.7)	23 (32.1)	162 (100)	< 0.002*
	Married	20 (33.3)	29 (48.3)	11 (18.3)	60 (100)	
	In a relationship	4 (8.5)	24 (51.1)	19 (40.4)	47 (100)	
Occupation	Student	23 (11.6)	107 (53.8)	69 (34.7)	199 (100)	< 0.001**
	Employed	24 (38.1)	27 (42.9)	12 (19.0)	63 (100)	
	Other	0 (0.00)	6 (85.7)	1 (14.3)	7 (100)	

*. Significant at the level of 5%

The results from Table 3 shows the association of different level of depression with some background characteristics. There is some indication of an insignificant association between depression and gender, Regio n, and family type. The level of depression remains nearly the same for both the male and female respondents, where 70% of both males and females face moderate to severe depression. On the other side, the association was found for the Relationship Status (*P*-value of ${\chi ^2}$= 0.000 < 0.05) and Occupation (*P*-value of ${\chi ^2}$= 0.000 < 0.05). About 80% of the people in a relationship are found to have moderate depression, whereas only about 38% of married people are facing so.

**Table 3:** Association between background characteristics and PHQ scale

Characteristics		Level of Depression				Total	*P*-value of ${\chi ^2}$
		Mild	Minimal	Moderate	Severe		
Gender	Male	17 (14.3)	19 (16.0)	72 (60.5)	11 (9.2)	119 (100)	0.776
	Female	19 (12.7)	23 (15.3)	88 (58.7)	20 (13.3)	150 (100)	
Region	Urban	32 (14.7)	36 (16.6)	122 (56.2)	27 (12.4)	217 (100)	0.167
	Rural	4 (7.7)	6 (11.5)	38 (73.1)	4 (7.7)	52 (100)	
Family Type	Nuclear	23 (12.6)	25 (13.7)	113 (61.7)	22 (12.0)	183 (100)	0.513
	Joint	13 (15.1)	17 (19.8)	47 (54.7)	9 (10.5)	86 (100)	
Relationship Status	Single	25 (15.4)	17 (10.5)	101 (62.3)	19 (11.7)	162 (100)	< 0.001*
	Married	9 (15.0)	23 (38.3)	22 (36.7)	6 (10.0)	60 (100)	
	In a relationship	2 (4.3)	2 (4.3)	37 (78.7)	6 (10.0)	47 (100)	
Occupation	Student	27 (13.6)	15 (7.5)	131 (65.8)	26 (13.1)	199 (100)	< 0.001*
	Employed	9 (14.3)	25 (39.7)	24 (38.1)	5 (7.9)	63 (100)	
	Other	0 (0.00)	2 (28.6)	5 (71.4)	0 (0.00)	7 (100)	

*. Significant at the level of 5%

Table 4 displays the association between the level of Facebook addiction and the level of depression measured from the BFAS and PHQ scale. A significant association between these two characteristics is found (*p*-value of ${\chi ^2}$= 0.000 < 0.05). As in Table 3, we can see about 70% of the participants who are identified as problematic Facebook users are in moderate depression, and more than 90% of the Facebook-addicted people are facing moderate to severe depression. On the other hand, only over 2% of the normal users are in severe depression, whereas nearly 70% of the normal users are in mild to minimal depression.

**Table 4:** Association between level of depression and level of Facebook use

Characteristics		Level of Depression				Total	*p*-value of ${\chi ^2}$
		Mild	Minimal	Moderate	Severe		
Level of Facebook Use	Normal Use	12 (25.5)	20 (42.6)	14 (29.8)	1 (2.1)	47 (100)	< 0.001*
	Problematic use	20 (14.3)	21 (15.0)	96 (68.6)	3 (2.1)	140 (100)	
	Facebook Addiction	4 (4.9)	1 (1.2)	50 (61.0)	27 (32.9)	82 (100)	

*. Significant at the level of 0.05

**Table 5:** Correlation matrix

Correlations		
	BFAS	PHQ
BFAS	1	0.701*
PHQ	0.701**	1

**. Correlation is significant at the 1% level (2-tailed)

The result of the ordinal regression is shown in Table 6, which displays the association of socio-demographical factors and activities of people with Facebook addiction and depression. From the tabulated result, we can observe female participants are at more risk of Facebook addiction and depression compared with their counterparts (*BFAS: β(95%CI) = .859(0.233,1.495);PHQ: β(95%CI) =.378(-0.252,1.007))*. Relationship status is also a significant factor in the case of depression *P value* < .05. The negative value indicates less risk of depression for married people (*BFAS: β(95%CI)* = .223(-1.424,0.978) than the people who are in a relationship. On the other hand, employed people are less likely to be addicted than the participants who are students *BFAS: β(95%CI)* = -0.494* (-1.547, 0.559)). Users who rarely post on Facebook are found to be less likely to be addicted to Facebook compared to the people who post daily (*BFAS: β(95%CI)* = -0.714(-1.543, 0.114)). It also shows that the employed people have a lower BFAS and PHQ score *BFAS: β(95%CI)* = -0.494* (-1.547, 0.559); *PHQ: β(95%CI)* = -0.601 (-1.65, 0.447)). Facebook users with desktops or laptops also have a significantly lower value of the estimate. The result also swowed that people who use Facebook mostly on desktops or laptops have a lower chance of Facebook addiction and depression. Compared to the students or unemployed, the employed persons are found less likely to be addicted to Facebook with an estimate of β(95%CI) = -0.494(-1.547, 0.559).

**Table 6:** Results of ordinal logistic regression

Factors		n (%)	BFAS $\beta$ (95% CI)	PHQ $\beta$ (95% CI)
Gender (ref: Male)	Female	150 (55.8)	0.859* (0.223,1.495)	0.378* (-0.252,1.007)
	Male	119 (44.2)		
Age (ref: 29+)	19–24	174(64.7)	-2.051*(-3.789, -0.313)	-0.125 (-1.703,1.452)
	25–29	57(21.2)	-1.18(-2.566,0.206)	-0.163 (-1.413,1.088)
	29+	38 (14.1)		
Resident area (ref: Rural)	Urban	217 (80.7)	-0.858* (-1.626, -0.09)	-0.209 (-0.932,0.514)
	Rural	52 (19.2)		
Family type (ref: Joint)	Nuclear	183 (68.0)	-0.125(-0.769,0.52)	0.709* (0.089,1.329)
	Joint	86 (32.0)		
Relationship status (ref: In a relationship)	Single	162 (60.2)	0.287(-0.482,1.057)	-0.756* (-1.524,0.013)
	Married	60 (22.3)	-0.223(-1.424,0.978)	-1.302* (-2.462, -0.142)
	In a relationship	47 (17.5)		
Occupation	Other	7 (2.6)	0.14(-2.159,2.439)	-0.25 (-2.086,1.586)
	Employed	63 (23.4)	-0.494* (-1.547,0.559)	-0.601 (-1.65,0.447)
	Student	199 (74.0)		
What kind of internet connection do you use	Both	190 (70.6)	-0.425(-1.206,0.355)	1.049* (0.279,1.818)
	Mobile Internet	38 (14.1)	-0.106(-1.097,0.886)	1.121* (0.134,2.108)
	Broadband	41 (15.2)		
How long have you been using Facebook	Less than 1 year	6 (2.2)	20.661(20.661,20.661)	1.049 (-0.904,3.002)
	1–2 years	11(4.1)	-1.095(-2.604,0.414)	1.409* (-0.148,2.965)
	2–5 years	89(33.1)	0.429(-0.273,1.131)	0.015 (-0.669,0.699)
	More than 5 years	163(60.6)		
Which device do you mostly use for Facebooking?	Desktop/laptop	10 (3.7)	-0.289(-1.782,1.203)	0.562 (-0.953,2.077)
	Mobile/Tab	259 (96.3)		
For which purpose do you use Facebook mostly	Socializing	99 (36.8)	-2.116(-5.057,0.825)	2.525* (-0.102,5.151)
	Entertainment	158 (58.7)	-2.327(-5.29,0.637)	2.859* (0.199,5.518)
	Academic	8 (3.0)	-2.274(-5.603,1.055)	2.498 (-0.511,5.507)
	Work	4 (1.5)		
How frequently do you post on Facebook	Rarely	168 (62.5)	-0.714(-1.543,0.114)	-0.006 (-0.806,0.794)
	At least once a week	62(23.0)	-0.871(-1.808,0.065)	0.409* (-0.498,1.316)
	At least once a day	39 (14.5)		
What do you post mostly on Facebook	Sharing incidents	79 (29.4)	-0.538(-1.516,0.439)	-0.062 (-1.028,0.904)
	Photos	104 (38.7)	-0.405(-1.336,0.527)	0.342 (-0.574,1.258)
	Funny contents	56 (20.8)	0.571 (-0.538,1.679)	-0.102 (-1.171,0.967)
	Academic and informative content	30 (11.2)		
What type of content do you like to see on Facebook?	Fitness	3 (1.1)	-0.637(-3.746,2.473)	0.457 (-2.471,3.385)
	Nutrition	11 (4.1)	0.176(-1.423,1.774)	-0.133 (-1.628,1.362)
	Entertainment	212 (78.8)	-0.175(-1.011,0.661)	-0.12 (-0.916,0.677)
	Others	43 (16.0)		
How many hours a day do you use Facebook	Less than 1 h	37 (13.8)	1.903* (0.684,3.121)	-1.318* (-2.508, -0.128)
	1–3 h	127 (47.2)	1.142* (1.151,3.132)	-0.842* (-1.801,0.117)
	3–6 h	77 (28.6)	2.288* (0.324,2.251)	-0.415 (-1.375,0.545)
	More than 6 h	28 (10.4)		
Approximately how many friends do you have on your social media account?	less than 200	38 (14.1)	-0.823(-2.42,0.774)	-0.027 (-1.536,1.483)
	200–1500	173 (64.3)	-0.666(-2.052,0.719)	0.055 (-1.223,1.334)
	1500–3000	45 (16.7)	-0.942(-2.398,0.515)	0.644 (-0.724,2.011)
	more than 3000	13 (4.8)		
Do you post a picture on FB or any other social media, then constantly check for the no. of reactions and comments?	No	73 (27.1)	0.267(-0.38,0.915)	-0.115 (-0.738,0.507)
	Yes	196 (72.9)		
Do you ever feel your working time has dragged down due to checking your social media feed?	No	64 (23.8)	-0.376(-1.075,0.323)	0.196 (-0.479,0.871)
	Yes	205 (76.2)		

*. Significant at the 5% level

## Discussion

The aim of the present study was to find out the factors associated with Facebook addiction and its impact on depression. Our study reveals, more than half of the people in this study are Problematic users, and 30% of Facebook users are addicted. These results are alarming in terms of the uncontrolled use of social media. The previous study showed that 39.7% Bangladeshi students were addicted in Facebook (Al Mamun & Griffiths, 2019). In an investigation from 2017 conducted in Southern India with graduate students from universities, Shettar et al. estimated a 26% Facebook addiction incidence with a threshold value of 15 employing the BFAS. According to data from the Thai-BFAS, the overall incidence percentage amongst high school students in Thailand was 41.8% when a threshold score of 12 or higher was used (Khumsri et al. 2015). In accordance with BFAS polythetic rating, a research investigation of students in Malaysia found that 47% of them had Facebook addiction (Jafarkarimi et al. 2016).

This study indicates almost the same ratio of female and male Facebook users though previous study showed more male users than female (Al Mamun & Griffiths, 2019). About 80.7% of people in this study are from an urban area, and this finding is not surprising given the rapid urbanization that has been taking place (Alam 2018). Also, large percent of Facebook users (70%) belong to a nuclear family which is also reasonable (Al Mamun & Griffiths, 2019). Importantly, the availability of smartphones plays a big role in using Facebook (Van Velthoven et al. 2018). This study showed almost all (96%) uses smartphones to use Facebook, and about 60% use it for entertainment. Though, most respondents (62.5%) rarely post on Facebook, and the rest post at least once a week or a day. Almost 80% of respondents feel that their working time has dragged down due to checking their social media feeds and still they are using it. This seems real as a previous study showed that the industrial and official productivity was found to be decreased as a result of using Facebook at workplaces (Lee and Lee 2018). Overall, the survey results suggest that most users access it through mobile devices and use it for some periods per day. However, association between Facebook addiction and socio demographic characteristics were evident from this study which contradicted the previous research conducted by Alenezi et al., in 2023 where they foud no association.

Through the PHQ scale, moderate depression was found among more than half of the participants, which is also a fact of concern. People often take Facebook as a way of passing stressed or depressed times or some dull moments (Brailovskaia et al. 2019). Normal Facebook users were found less depressed when the level of depression increased with the use of Facebook. Where most of the normal users were in mild and minimal depression there were more than 90% of the Facebook addict were in moderate to severe depression. Furthermore, Facebook addiction and depression was moderately correlated (0.701). The results of the current Bangladeshi study are consistent with previous investigations conducted overseas in Bangladesh which found links amongst Facebook addiction along with depression (Błachnio et al. 2015; Pantic et al. 2012; Shensa et al., 2017; Wright et al. 2013).

From the result of ordinal regression, we observe female participants are at more risk of Facebook addiction and depression compared with their counterparts, this is obvious as females are generally less involved with outer world (Alenezi et al., 2023). This finding also supported the result of Cudo et al. 2020. Compared to the students or unemployed, the employed persons are found less likely to be addicted to Facebook which is understandable since employed persons remain busy with their work and spend less time on Facebook (Al Mamun & Griffiths, 2019). The newer users were at more risk of being in depression and getting addicted which seems very usual. Since new users can not understand the limit of a new thing. The participants who were using Facebook for less than 2 years were found in more risk to get depression, and those who had been using it for less than 1 year had a very high risk of Facebook addiction. The relationship of what people post on Facebook with the addiction level was also found to be significant. The people who post funny content on Facebook are more likely to be addicted compared to those who share academic and informative content. Facebook usage time in a day was also a proactive factor for higher depression and addiction.

## Conclusion

The research brings the distribution of Facebook usage of people of different age range, region, occupation and others. The relationship between Facebook usage and depression is analysed in this study and found the presence of association. Relationship between Facebook usage, depression are also studied and there correlation matrix is presented. It also reveals the factors that are significantly influencing the Facebook addiction to the people. As it is found from this research that mental helath is associated with the useage of Facebook, the factors those are responsible for the addiction to be controlled to have a better and depression free life. The arrangement of more mental health campaign can help to understand facebook users about the bad impact of its use and also can provide a guideline of bounded and legitimate use of facebook. In order to enhance their mental health, the individual who is moderately or severely depressed should also have psychological assistance. To get better, Facebook addicts should also get the appropriate clinical counselling.

## Strength and limitations

The main strength of this study is no authors have investigated Facebook addiction and depression among general people of Bangladesh. To the best knowledge of authors, this is the first study where large number of predictors were considered to make the link aamong Facebook addiction and depression. Our study also have some limitations. Firstly, the samples are limited and was taken using convenience sampling procedure and the sample size is small. The study can be repeated after enlarging the sample size. Secondly, only self-reported facebook use was assessed here which may contains reporter bias. Therefore, the result of this study may not generalized overall picture of the society. Here, other potential influencer was not considered for instance, social support and adjusting strategies on the association between usage of facebook and depression. These shortcomings may have an impact on the study’s finding’s validity. Also, It is impossible to determine whether the respondent is depressed due to other problems, such as familial problems.

Furthermore, longitudinal study is needed in order to determine if the variables that are dealt with in this study are also linked not only depression but also other psychiatric comorbidities such as anxiety, personality disorders, loneliness, stress, and self-esteem etc.

## Data Availability

The data will made available at anytime if requested.
